# Supplementary material for: A modular framework for multi-scale tissue imaging and neuronal segmentation
Source: Nat Commun. 2024 May 22;15:4102. doi: 10.1038/s41467-024-48146-y (PMC11111705; doi:10.1038/s41467-024-48146-y)
Supplement: Supplementary file 1 — Supplementary Information [file 41467_2024_48146_MOESM1_ESM.pdf]

Supplementary Information

Supplementary Note 1. Available Algorithms

We report in Supplementary Table 1 a list of automatic segmentation and tracing algorithms, from the last two reviews on the topic[1,2]. For methods based on deep learning, we refer to Chen et al., 2023[3].

Supplementary Table 1. List of automatic segmentation/tracing algorithms adapted from Liu et al., 2022[2], Magliaro et al., 2019[1].

Algorithm	Authors	doi
**ViterBrain**	Athey et al. (2022)	10.1038/s42003-022-03320-0
**RPCT**	Bas and Erdogmus (2011)	10.1007/s12021-011-9105-2
**SmartTracing**	Chen et al. (2015)	10.1007/s40708-015-0018-y
**Neural Circuit Tracer**	Chothani et al. (2011)	10.1007/s12021-011-9121-2
**Active learning of neuron morphology for accurate automated tracing of neurites**	Gala et al. (2014)	10.3389/fnana.2014.00037
**CAAT**	Huang et al. (2021)	10.3389/fnana.2021.712842
**ORION2**	Jiménez et al. (2015)	10.1007/s12021-014-9256-z
**ShuTu**	Jin et al. (2019)	10.3389/fninf.2019.00068
**SparseTracer**	Li et al. (2017)	10.1371/journal.pone.0182184
**Rivulet2**	Liu et al. (2018c)	10.1109/TMI.2018.2833420
**ORION**	Losavio et al. (2008)	10.1152/jn.90627.2008
**flNeuronTool**	Ming et al. (2013)	10.1371/journal.pone.0084557
**APP1**	Peng et al. (2011)	10.1093/bioinformatics/btr237
**UltraTracer**	Peng et al. (2017)	10.1038/nmeth.4233
**NeuroGPS-Tree**	Quan et al. (2016)	10.1038/nmeth.3662
**PHD**	Radojević and Meijering (2017a)	10.1093/bioinformatics/btw751
**PNR**	Radojević and Meijering (2019)	10.1007/s12021-018-9407-8
**NeuronStudio**	Wearne et al. (2005)	10.1016/j.neuroscience.2005.05.053
**Open-Curve Snake**	Wang et al. (2011)	10.1007/s12021-011-9110-5
**ENT**	Wang et al. (2017)	10.1007/s12021-017-9325-1
**DiMorSC**	Wang et al. (2018)	10.1101/321489
**MOST**	Wu et al. (2014)	10.1016/j.neuroimage.2013.10.036
**APP2**	Xiao and Peng (2013)	10.1093/bioinformatics/btt170
**SimpleTracing**	Yang et al. (2013)	10.1186/1471-2105-14-93
**FMST**	Yang et al. (2019)	10.1007/s12021-018-9392-y
**MDL constrained 3-D grayscale skeletonization**	Yuan et al. (2009)	10.1007/s12021-009-9057-y
**neuTube**	Zhao et al. (2011)	10.1007/s12021-011-9120-3
**Neuron Crawler**	Zhou et al. (2015b)	10.1109/ISBI.2015.7164009
**TReMAP**	Zhou et al. (2016)	10.1007/s12021-015-9278-1
**neurolucida**	Glaser et al. (1990)	10.1016/0895-6111(90)90105-k
**mansegtool**	Magliaro et al. (2017)	10.3389/fninf.2017.00036
**tree2tree**	Basu et al. (2013)	10.1109/TITB.2012.2209670
**trees**	Cuntz et al. (2010)	10.1371/journal.pcbi.1000877
**g-cut**	Li et al. (2019)	10.1038/s41467-019-09515-0

Supplementary Note 2. Labeling and super-resolution STED imaging of thick samples

A movie showing 3D rotating views of acquired standard- and super-resolution datasets is supplied as Supplementary File (Supplementary Movie 1).

Supplementary Note 3. Segmentation on dense standard-resolution confocal stacks

A movie showing 3D rotating views of a dense 40x confocal stack (red) and one segmented neuron (green) is supplied as Supplementary File (Supplementary Movie 2).

Supplementary Note 4. Stability of image topology encoding across different images

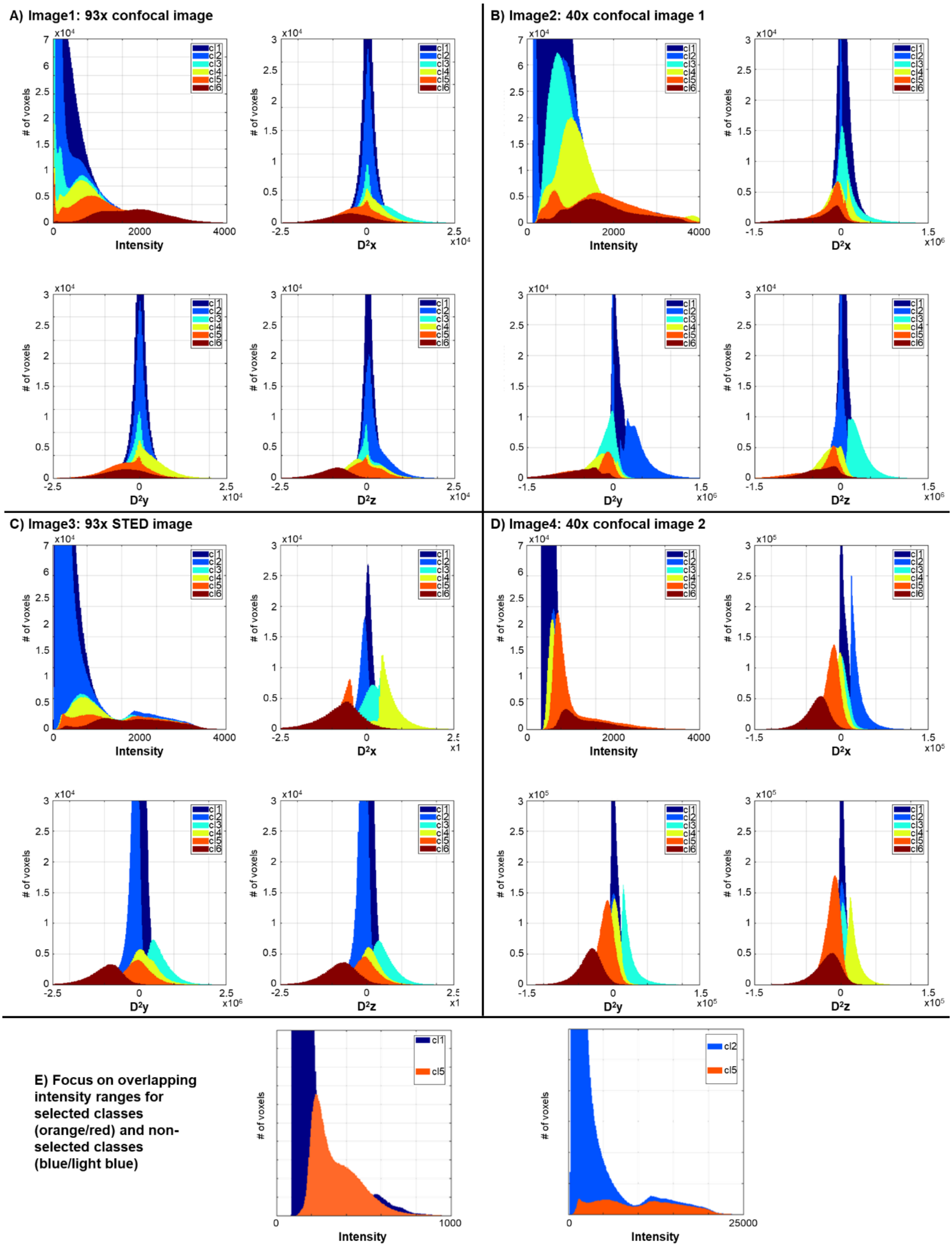

Supplementary Figure 1 – Histograms for intensity and second-order derivatives across K-means classes. **A-D)** For 4 different images (two 40x confocal images, one 93x confocal image and one 93x STED image), we report the histograms for class subdivisions as determined by the K-means performed on the 4-dimensional space including intensity and the three second-order derivatives computed along the three main axes. **E)** A zoom on the intensity histograms of only one selected class (warm colors) and one non-selected class (cold colors): we observe that the two covered ranges overlap in both cases, with the non-selected class including also high-intensity pixels and the selected class including also low-intensity ones.

Supplementary Note 5. Additional Comparisons across segmentation/tracing tools

We report in Supplementary Figures 2, 3, and 4, the outcomes of SENPAI, HK-Icy, NeuTube, Ilastik and NeuroGPS. The metrics of such segmented – or traced- neurons are reported in Figures 4 and 5 of the manuscript. Even though SENPAI provides the volumetric reconstruction of the neurons, their tracings can be easily achieved, to facilitate the visual and quantitative comparison among tools. The tracing can be performed with the skeletonization algorithms available in literature, or with the functions included in SENPAI. Here, the latter was used to skeletonize the volumetric segmentations provided by SENPAI and HK-Icy.

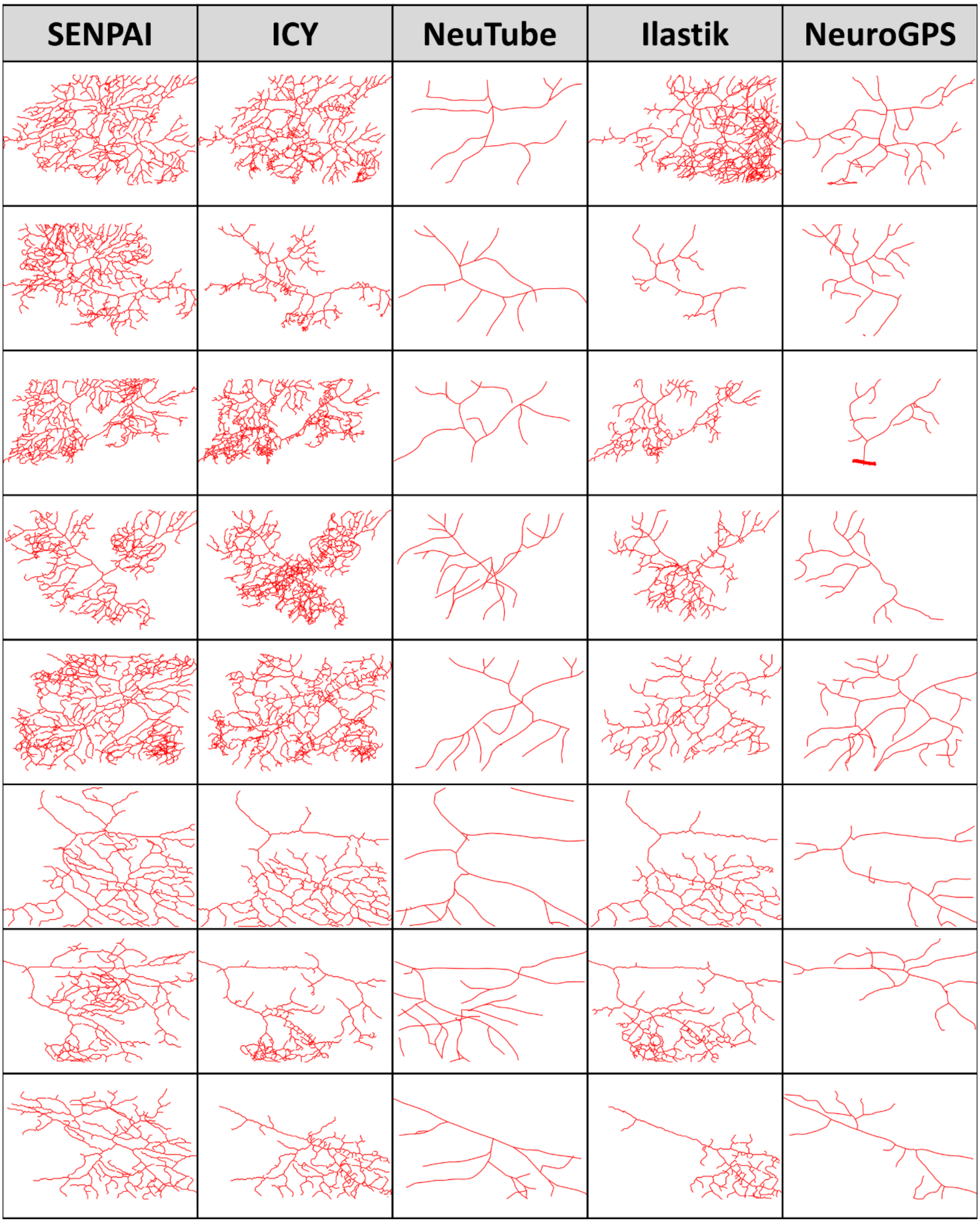

Supplementary Figure 2 – Examples of tracings obtained on standard-resolution images – part I. We report 3D views of the tracings for 9 neurons from the two 40x datasets, obtained with SENPAI (1st column), HK-Icy (2nd column), NeuTube (3rd column), Ilastik (4th column) and NeuroGPS (5th column). For SENPAI and HK-Icy, the segmentation was converted to a skeleton with a custom code provided within SENPAI.

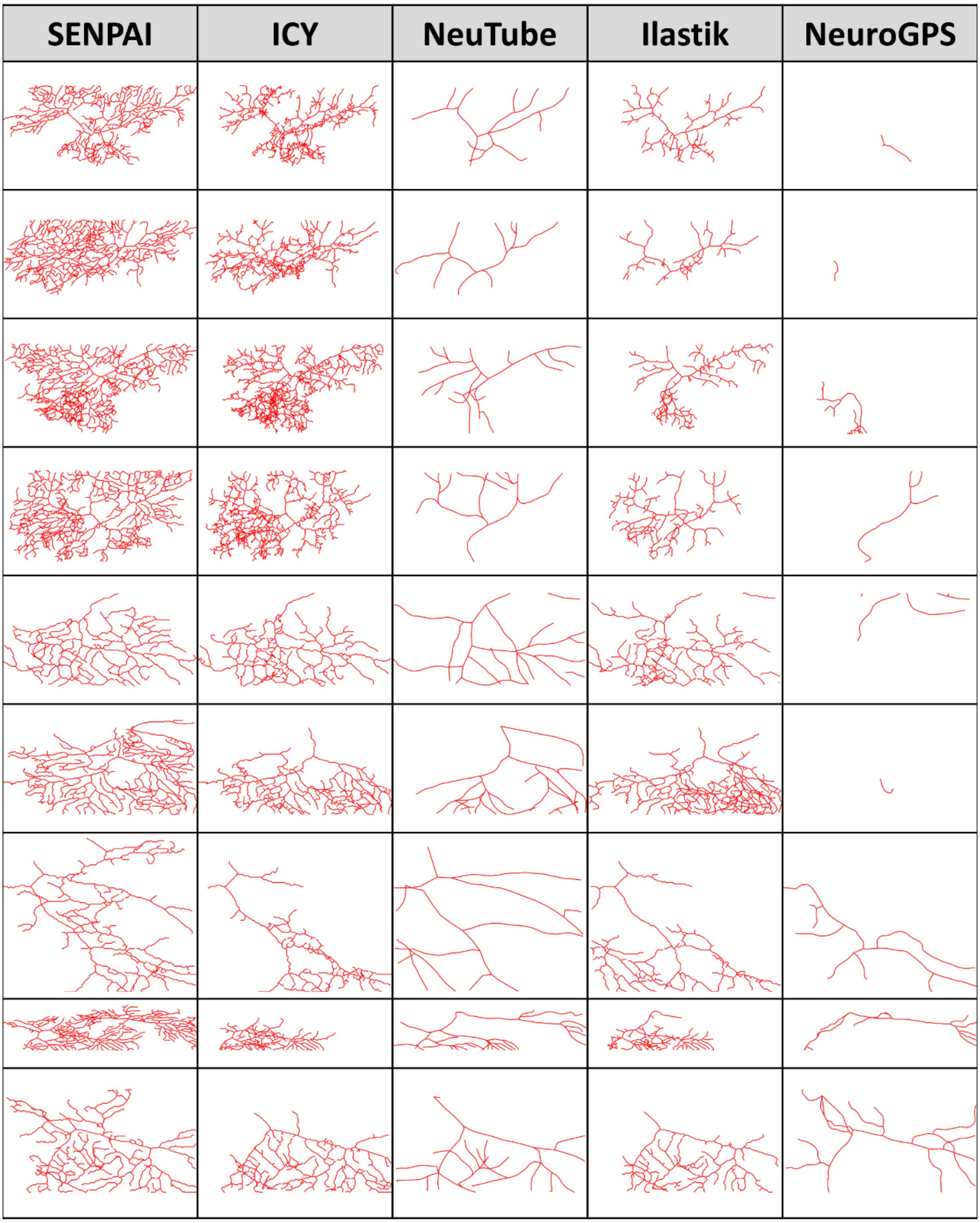

Supplementary Figure 3 – Examples of tracings obtained on standard-resolution images – part II. 3D views of the tracings for 9 neurons from the two 40x datasets, obtained with SENPAI (1st column), HK-Icy (2nd column), NeuTube (3rd column), Ilastik (4th column) and NeuroGPS (5th column). For SENPAI and HK-Icy, the segmentation was converted to a skeleton with a custom code provided within SENPAI.

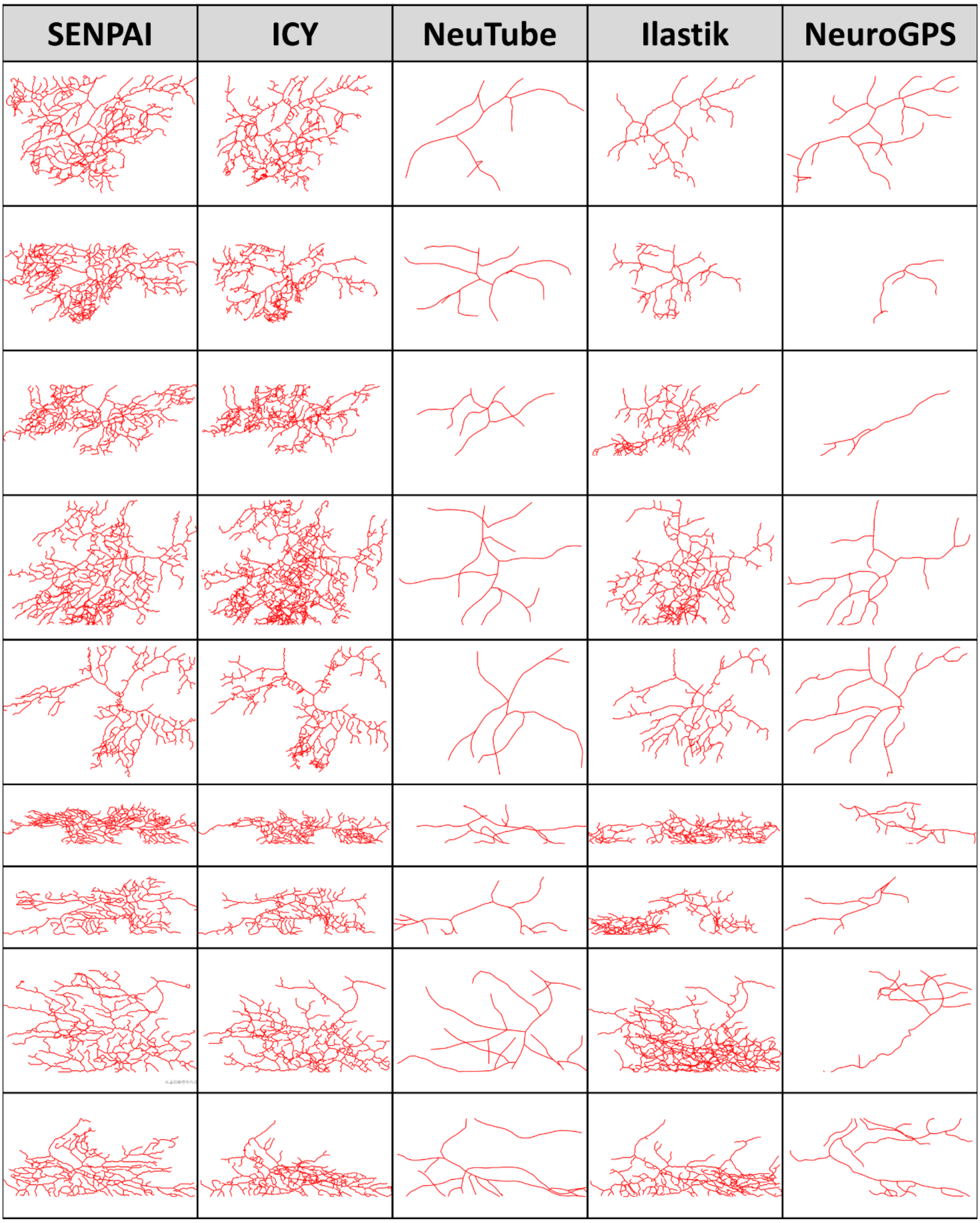

Supplementary Figure 4 – Examples of tracings obtained on standard-resolution images – part III. We report 3D views of the tracings for 9 neurons from the two 40x datasets, obtained with SENPAI (1st column), HK-Icy (2nd column), NeuTube (3rd column), Ilastik (4th column) and NeuroGPS (5th column). For SENPAI and HK-Icy, the segmentation was converted to a skeleton with a custom code provided within SENPAI.

Supplementary Note 6. Segmentations of single neurons with the HK-Means plugin of Icy: targeting neuron separation

The results obtained with HK-Icy derive from several tests performed with different setting parameters (number of intensity classes, minimum and maximum cluster size, minimum intensity value). As stated in the main text, the target was the maximization of the segmentation density without losing its tree-like structure. Previous attempts were conducted by maximizing the density of the dendritic tree detected by the algorithm, while assuring that each fully-connected segmentation cluster identified one single neuron, i.e., not allowing for clusters connecting different neurons. This previous criterion resulted in a visible under-segmentation: results are reported in Supplementary Figures 5 and 6. Such figures report the segmentations obtained with SENPAI - gray mesh-, and the neuron cluster segmentations obtained with HK-Icy, touching the segmentation obtained for the same neuron with SENPAI. In fact, the segmentations obtained with HK-Icy also presented smaller clusters, identifying smaller branches that could not be identified as connected to a soma. These figures show the impossibility for HK-Icy to obtain on our standard resolution datasets a single neuron reconstruction providing a sufficient amount of detail of the neuron structure, thus motivating our efforts in embedding the watershed-based approach presented in our algorithm.

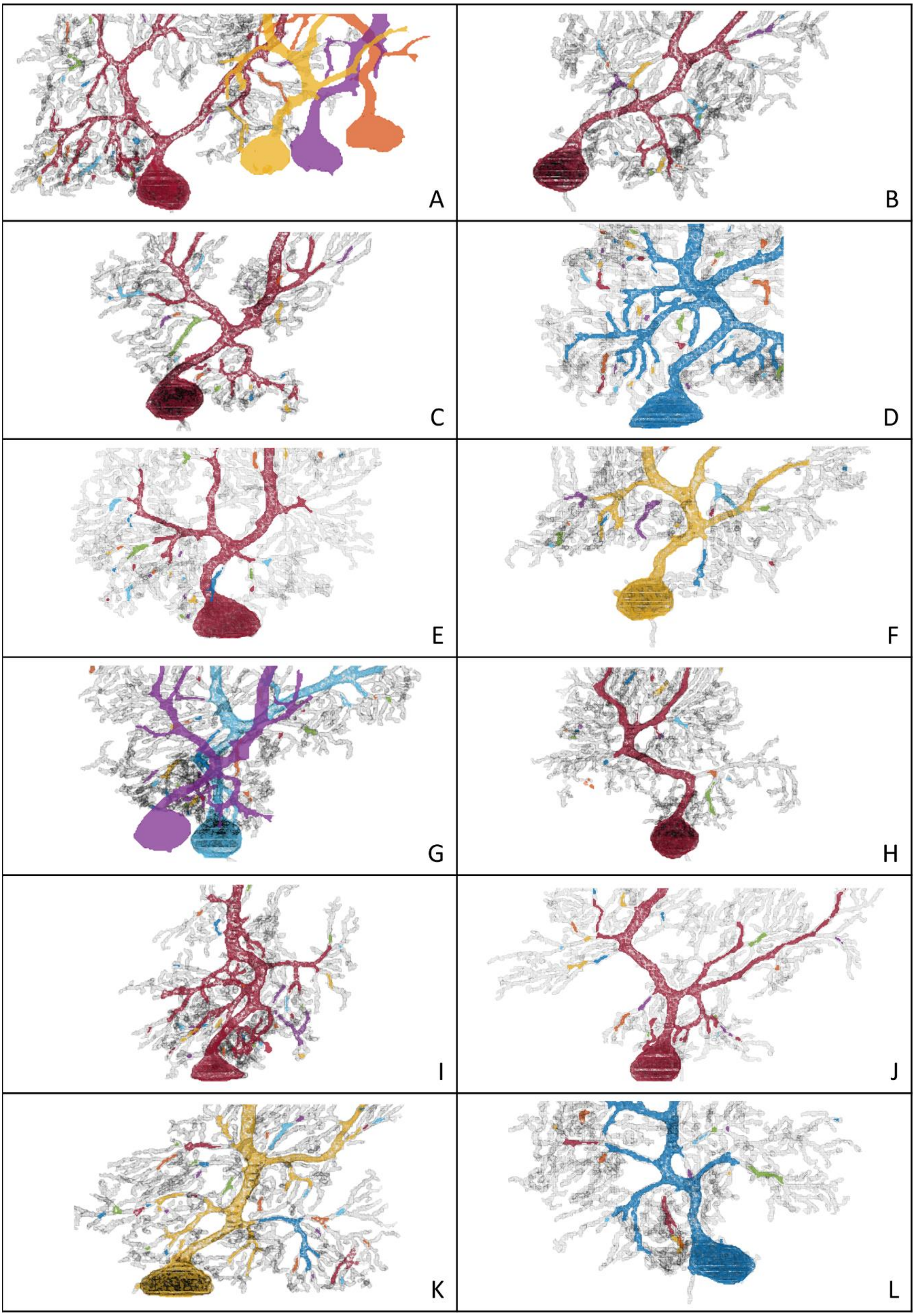

Supplementary Figure 5 – Groups of clusters in the HK-Icy segmentation touching the same SENPAI segmentation. For 12 neurons of a confocal dataset representing PC from clarified murine cerebellar slice, we report the SENPAI segmentation – gray mesh -, and the disconnected HK-Icy segmentations in contact with the specific SENPAI outcome -colored meshes -.

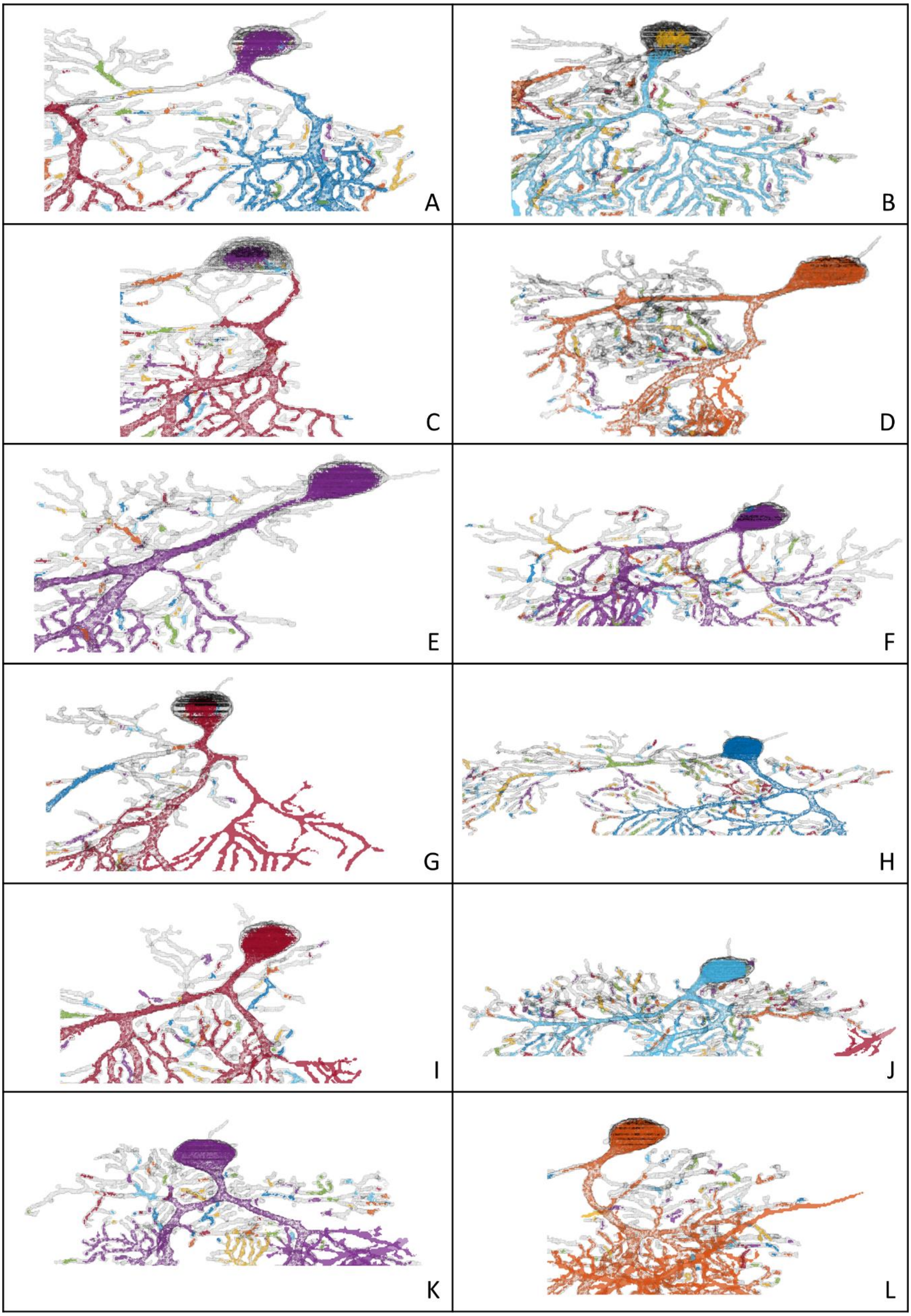

Supplementary Figure 6 – Groups of clusters in the HK-Icy segmentation that touch the same SENPAI segmentation. For 12 neurons of the hor512 dataset we report with a gray mesh the SENPAI segmentation and with colored clusters the pieces of disconnected HK-Icy segmentations that are in contact with it.

Supplementary Note 7. Segmentations of single neurons with the HK-Means plugin of Icy: targeting arborization density

To obtain denser segmentations using the HK-Means plugin of Icy, we used step 2 of the SENPAI pipeline to parcellate the segmentation of multiple neurons. To this end, the most impacting parameter is the Maximum Object Size (MOS). On one standard resolution dataset, using 4 classes, with MOS=50000, every segmented cluster is associated with one single neuron (see Supplementary Figures 5 and 6). When the Maximum Object Size is increased, observed effects are: 1) an increase in the density and complexity of the resulting clusters; 2) the presence of connected clusters including multiple neuronal structures. By increasing the MOS, we observed a sharp increase of the segmentation density at a specific MOS (Supplementary Figure 7-A). The same phenomenon is observed on two different datasets and with three different values for the number of classes (#CL). The segmentation obtained with a MOS value right above the 'knee', is the one that was observed to maximize density while preserving the tree-like structure of arborizations. Nonetheless, with respect to SENPAI segmentation, over-segmentation seems to occur in the brightest slices of the image, while under-segmentation seems to occur at the darkest ones (Supplementary Figure 7-B). On the other hand, HK-Icy seems to be particularly sensitive to intensity inhomogeneity along the z-axis. In Supplementary Figure 5-C, we report the 3D comparisons for two neurons as segmented with SENPAI and with HK-Icy, with the selected parameters. It is possible to observe enhanced details of the dendritic trees where the inhomogeneity of the segmentation obtained with HK-Icy is particularly evident.

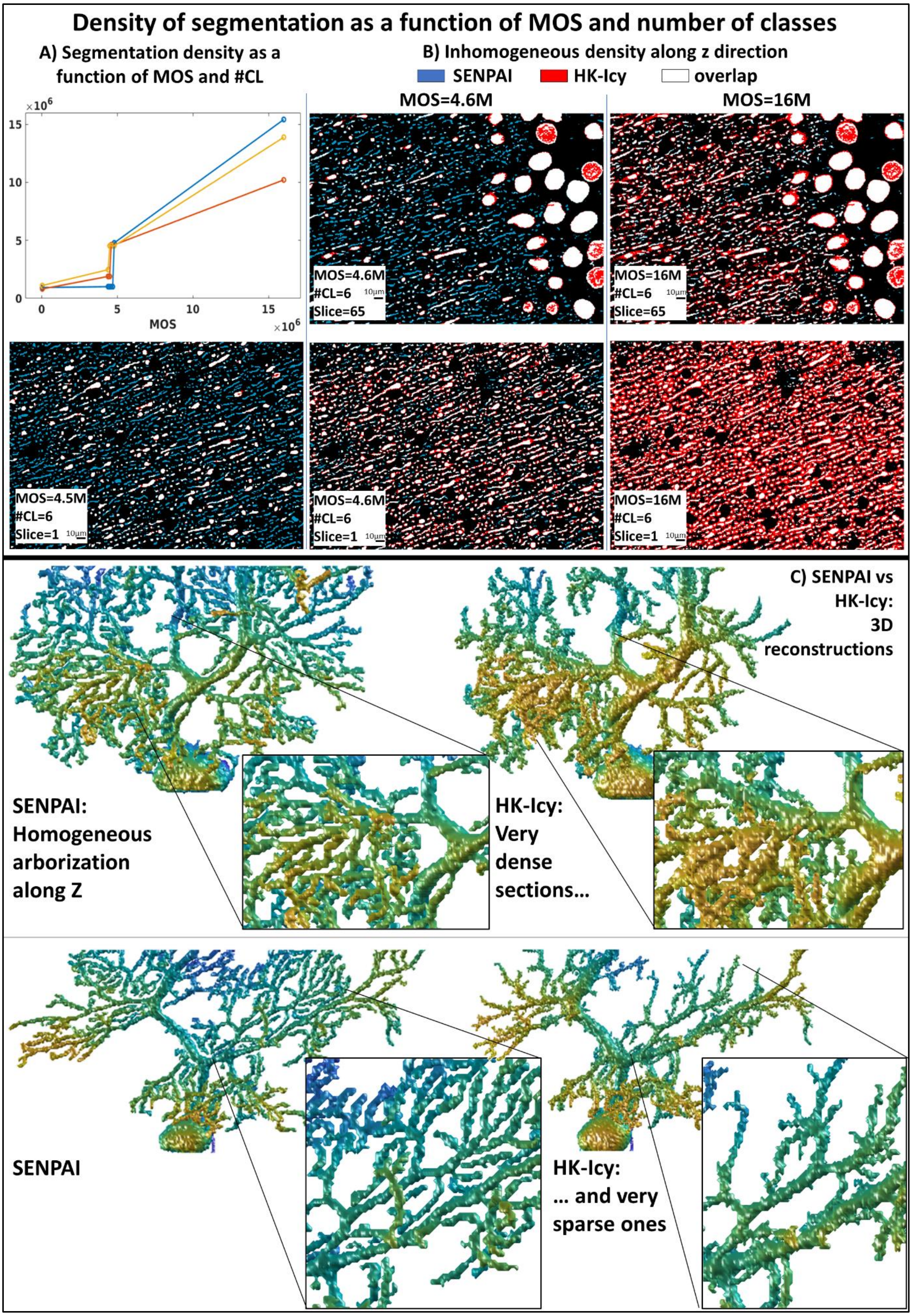

Supplementary Figure 7 – Segmentation parameters in HK-Icy: A) Segmentation density (number of segmented voxels to total number of voxels ratio) as a function of the maximum object size (MOS), for 3 numbers of classes #CL (#CL=4 blue, #CL=6 red, #CL=8 yellow); the ‘knee’ is clearly visible for all of the three lines. B) The densest slice for HK-Icy segmentation obtained

with #CL=6, MOS=4.5M (slightly below the knee), along with SENPAI segmentation, is shown on the left; For MOS=4.6M (slightly above the knee, middle) and MOS=16M, we compare a middle slice (slice 65, top) and the first slice (bottom) for HK-Icy and SENPAI. C) 3D comparisons between SENPAI segmentations of single neural structures (left), and the same neuron as segmented by HK-Icy with #CL=6 and MOS=4.6M; zoomed details show differences between the two algorithms in terms of density homogeneity.

Supplementary Note 8. Custom code for Strahler Analysis

To define a Strahler Ordering on the segmentations obtained with SENPAI and Icy, we used the skeletonization command already available in Matlab. Information on nodes diameters was extracted by computing the distance from segmentation edges at the nodes' coordinates. This was done by exploiting the distance transform computed on the complement of the segmentation. The skeleton was converted into a graph object by defining an edge between each pair of neighboring voxels in the skeleton. Then, any cycles were detected and avoided by removing the edge at minimum intensity and minimum diameter. The acyclic graph was converted to a minimum spanning tree, defining as root the skeleton node closest to the mask of somas but not belonging to it. The tree is defined by a list of node indexes, their coordinates, and a vector of predecessor nodes, specifying for each node the index of its parent. From this list we extracted a list of leaf nodes (nodes with no children) and of bifurcation nodes (nodes with more than one child).

Strahler orders are assigned to edges as follows: a weight of 0.5 is assigned to each weight in contact with a bifurcation node, and 0 to all the other edges. For each node, the Strahler order of the segment it belongs to is defined by the round towards minus infinite of the length of the shortest path tree between the node and the root.

Segments are defined by a neighboring set of nodes enclosed between two special nodes (i.e., bifurcations, leaves or root nodes). For each segment, the length is defined as the sum of Euclidean distances between connected nodes, while the diameter is defined as the average diameter across nodes.

Segments are grouped into branches by climbing the whole tree iteratively starting from each leaf, looking for neighboring segments with parent-child relation and sharing the same Strahler Order. The length and diameter of each branch are computed in the same way as for segments.

The SN is extracted as the maximum SO presented by the segments of a neuron. Th Normalized Number of Segments per SO k is simply computed as the number of segments for each SO, divided by the total number of segments. The same is done with branches to compute the Normalized Number of Branches per SO k. To both sets of values we fit a linear polynomial in the binary semi-logarithmic space and we extract the slope and the determination coefficient R^2. For the Branch Bifurcation Ratio, we plot the differential of the number of Branches in the logarithmic space and we perform a linear fit in the linear one to extract the slope and the coefficient of determination R^2. To compute the Topological Subtree Size for each branching node, we count for each branching node in the tree at SO k, the number of bifurcation nodes and leaf nodes in its children subtree.

Supplementary Note 9. Strahler-Ordering-based Validation: Results

We compare the features described in Vormberg et al. (2017)[4] with those extracted from our segmentations. SENPAI and HK-Icy achieved segmentations with the same SN mode identified by Vormberg et al. (2017) for Purkinje cells, with SENPAI showing considerably more hits (in brackets). Considering the normalized number of segments, SENPAI neurons matched the steepest decay (slope=-1.20) found for Purkinje cells in the reference work. The curve of HK-Icy is similar but displays larger deviations, as happens for the subtree size and for the total dendritic length as well. Differences across algorithms were very small in the case of the slope for the normalized number of branches, with all of them achieving values in the reference range or just slightly above it. SENPAI, HK-Icy and NeuTube provide Purkinje-like Branch Bifurcation Ratio. For the topological subtree size, we observe slopes all slightly below the range reported by Vormberg and co-workers. This was particularly true for HK-Icy and SENPAI. Regarding the total dendritic length and the slope of the normalized average branch length per SO k, only SENPAI and HK-Icy show values in the range of the reference work. We highlight the fact that for two measures out of the three that are recognized as relevant for neuron type discrimination in Vormberg et al, SENPAI outperforms the other algorithms in terms of similarity with the Purkinje cells.

Feature	Vormberg et al. 2017	SENPAI, HK-Icy, NeuroGPS, NeuTube
**Strahler Number Distribution across neurons**	Purkinje: mode=6 (11/14)	SENPAI: mode=6 (15/27)<br>HK-Icy: mode=6 (11/27)<br>NeuroGPS: mode=2 (10/27)<br>NeuTube: mode=3 (19/27)<br>Ilastik: mode=5 (14/27)
**A) Normalized Number of Segments per SO k: Linear fit slopes in the binary logarithmic space**	Range [-1.20, -0.89] (R^2 > 0.9686)<br>(Approximates 2^{-k} in the linear space, steepest slope for Purkinje)	SENPAI=-1.23 (R^2=0.9887)<br>HK-Icy=-0.99 (R^2=0.9964)<br>NeuroGPS=-0.86 (R^2=1.0000)<br>NeuTube=-0.90 (R^2=0.9983)<br>Ilastik=-1.14 (R^2=0.9864)
**B) Normalized Number of Branches per SO k: Linear fit slopes in the binary logarithmic space**	Range [-0.54, -0.45] (R^2 > 0.996)<br>(Approximates 4^{1-k} in the linear space)	SENPAI=-0.47 (R^2=0.9961)<br>HK-Icy=-0.44 (R^2=0.9953)<br>NeuroGPS=-0.47 (R^2=1.0000)<br>NeuTube=-0.44 (R^2=0.9744)<br>Ilastik=-0.49 (R^2=0.9978)
**C) Branch Bifurcation Ratio: Linear fit slopes**	Range [2.23, 3.77]	SENPAI=3.55 (R^2=0.9909)<br>HK-Icy=3.73 (R^2=0.9606)<br>NeuroGPS=4.27 (R^2=8280)<br>NeuTube=3.01; (R^2=0.7342)<br>Ilastik=3.69 (R^2=0.9816)
**D) Normalized Topological Subtree Size: Linear fit slopes in the logarithmic space**	Range [0.54, 0.65] (R^2 > 0.9898)<br>(Approximates 4^{k-SN} in the linear space)	SENPAI=0.47 (R^2=0.9972)<br>HK-Icy=0.43 (R^2=0.9958)<br>NeuTube=0.48 (R^2=1.0000)<br>NeuroGPS: SN too low to perform the regression.<br>Ilastik=0.48 (R^2=0.9934)
**E) Normalized Branch Diameter per SO k: R^2 for the approximation to k^2**	R^2 > 0.9956	SENPAI=0.8511<br>HK-Icy=0.2201<br>NeuroGPS=1.0000<br>NeuTube=0.9989<br>Ilastik=0.8657
**F) 1 - Total Normalized Dendritic Length per SO k: Linear fit slopes in the semi-logarithmic space**	Range [-0.31, -0.76] (R^2 > 0.9892)<br>(Approximating an exponential decay)	SENPAI=-0.35 (R^2=0.9961)<br>HK-Icy=-0.31 (R^2=0.9872)<br>NeuroGPS=-0.21 (R^2=1.0000)<br>NeuTube=-0.26 (R^2=0.9416)<br>Ilastik=-0.34 (R^2=0.9923)
**F) 2 - Total Normalized Dendritic Length per SO k: Percentage of the total dendritic length in SO 1**	50 to 60% for planar morphologies (e.g., Purkinje), above 80% for 3D-distributed morphologies	SENPAI=52%<br>HK-Icy=55%<br>NeuroGPS=62%<br>NeuTube=57%<br>Ilastik=53%
**G) Normalized Average Segment Length per SO k: Linear fit slopes in the semi-logarithmic space**	Approximately constant for planar morphologies (e.g., Purkinje), negative slopes in the range [-0.45 -0.17] for 3D-distributed morphologies	SENPAI=0.0225 (R^2=0.5755)<br>HK-Icy=-0.0202 (R^2=0.6759)<br>NeuroGPS=0.0074(R^2=0.2234)<br>NeuTube=0.0303 (R^2=1.0000)<br>Ilastik=0.0016 (R^2=0.0077)
**H) Normalized Average Branch Length: Linear fit slopes up to the second-to-last SO in the semi-logarithmic space**	Range [0.18 0.30] for planar morphologies (e.g., Purkinje), nearly constant or decreasing for 3D-distributed morphologies	SENPAI=0.22 (R^2=0.9472)<br>HK-Icy=0.20 (R^2=0.9429)<br>NeuroGPS and NeuTube: SN too low to perform regressions<br>Ilastik=0.33 (R^2=0.9916)

Supplementary Table 2 – Numerical comparison of the SO of Purkinje cells found in literature and measured on the neurons segmented with SENPAI and the state-of-art tools. The 8 parameters based reported in Vormberg et al. 2017 (first column) for Purkinje cells (second column) and computed on 27 neurons segmented from the 40x datasets with SENPAI, HK-Icy, NeuroGPS and NeuTube (third column). The values in the third column in accordance with the ranges in the second column are underlined.

Supplementary Note 10. SENPAI's benchmarking against state-of-the-art algorithms on independent datasets using the BigNeuron resource

We exploited BigNeuron (Manubens-Gil et al., 2023[5]) to test the efficacy of SENPAI on independent datasets and against previously published neuron segmentation tools. BigNeuron is a resource to benchmark and predict the performance of algorithms for automated tracing of neurons in light microscopy datasets whose details are available in Manubens-Gil et al., 2023[5]. Among the features available in the BigNeuron resource, the authors provide (i) a database with gold-standard manual segmentations available, (ii) a set of algorithms for obtaining the neuron tracing, (iii) a set of metrics for comparing different algorithms, and (iv) an R-based Shiny app to compare new segmentations (in our case, the segmentations provided by SENPAI) of the benchmark datasets with the gold standard manual segmentation and against other algorithms.

Here, we focused on a subset of the 3D image volumes of the Gold166 dataset (http://web.bii.a-star.edu.sg/bigneuron/gold166.zip) made of datasets of mammalian brain cells. Particularly, we segmented neurons from the (i) human Allen (confocal datasets of pyramidal neurons), (ii) human cultured (confocal datasets of excitatory neurons stem-cell derived), (iii) mouse cultured (two-photon imaging datasets of excitatory neurons) and (iv) mouse RGC (confocal datasets of retinal ganglion cells) subsets. For each subset, we report all the available metrics as reconstruction quality indexes: (i) the entire-structure-average-from-gold standard-to-neuron (ii) the entire-structure-average-from-neuron-to-gold standard, (iii) the average of bidirectional entire structure averages with respect to the gold standards, (iv) the different structure average, (v) the percent of different structure from gold standard to neuron, (vi) the percent of different structure from neuron to gold standard,(vii) the percent of different structure and (viii) the aggregated distance[5,6]. Finally, we exploited the BigNeuron Shiny app – in particular standalone version of the app on a system running Ubuntu 22.04 - to compare the SENPAI reconstructions with those obtained from state of the art algorithms. Of note, the BigNeuron R-based shiny app for comparing algorithms is available both as standalone application, as well as a web app. Here, we exploited the standalone version of the app in a system running Ubuntu 22.04 for comparing the segmentations. Indeed, among the many features available in the shiny app, the comparison of different segmentations can be performed only locally, in a machine running Ubuntu and with Vaa3d already installed.

Figures from S8 to S16 report an example of the SENPAI segmentation of a cell from the human Allen confocal dataset, along with the result of the benchmark for each considered feature on the four subsets.

We observed that SENPAI performed well, with comparable or better performance with respect to most popular state of the art algorithms (Advantra, Annotated, app1, app2, app2new1, app2new2, app2new3, axis_analyzer, Consensus, Cwlab_ver1, ENT, EnsembleNeuronTracerBasic, EnsembleNeuronTracerV2n, EnsembleNeuronTracerV2s, fastmarching_spanningtree, LCMboost, LCMboost_3, meanshift, MOST, MST_Tracing, nctuTW, nctuTW_GD, NeuroGPSTree, NeuronChaser, NeuroStalker, neutu_autotrace, neutube, pyzh, Rayshooting, Rivulet, Rollerball, simple, smartTracing, snake, tubularity_model_S, XY_3D_TreMap). In this light, we emphasize that although SENPAI was developed for dealing with clarified samples representing dense-packed neurons, it still shows good performance on independent benchmark available in the state of art.

For each metric we summarize the ranking of the SENPAI outcome among all the considered algorithms:

(i) the entire-structure-average-from-gold standard-to-neuron (2/35)

(ii) the entire-structure-average-from-neuron-to-gold standard (3/35)

(iii) the average of bidirectional entire structure averages with respect to the gold standards (1/35)

(iv) the different structure average (1/35)

(v) the percent of different structure from gold standard to neuron (11/35)

(vi) the percent of different structure from neuron to goldstandard (11/35)

(vii) the percent of different structure (8/35)

(viii) aggregated distance (3/35)

Metrics (i), (ii), (iii) and (viii) measure how different the two reconstructions are. Metrics (iv), (v), (vi), (vii) measure the extent of differences between two reconstructions considering only points above a tolerance threshold S (here set to the default value of 2 voxels, as implemented in Vaa3d and in the Bigneuron resource) (Peng et al 2011).

SENPAI performed extraordinarily well for both types of metrics, ranking among the best algorithms for metrics (i), (ii), (iii), (iv), (viii) and satisfactorily well for metrics (v), (vi), (vii).

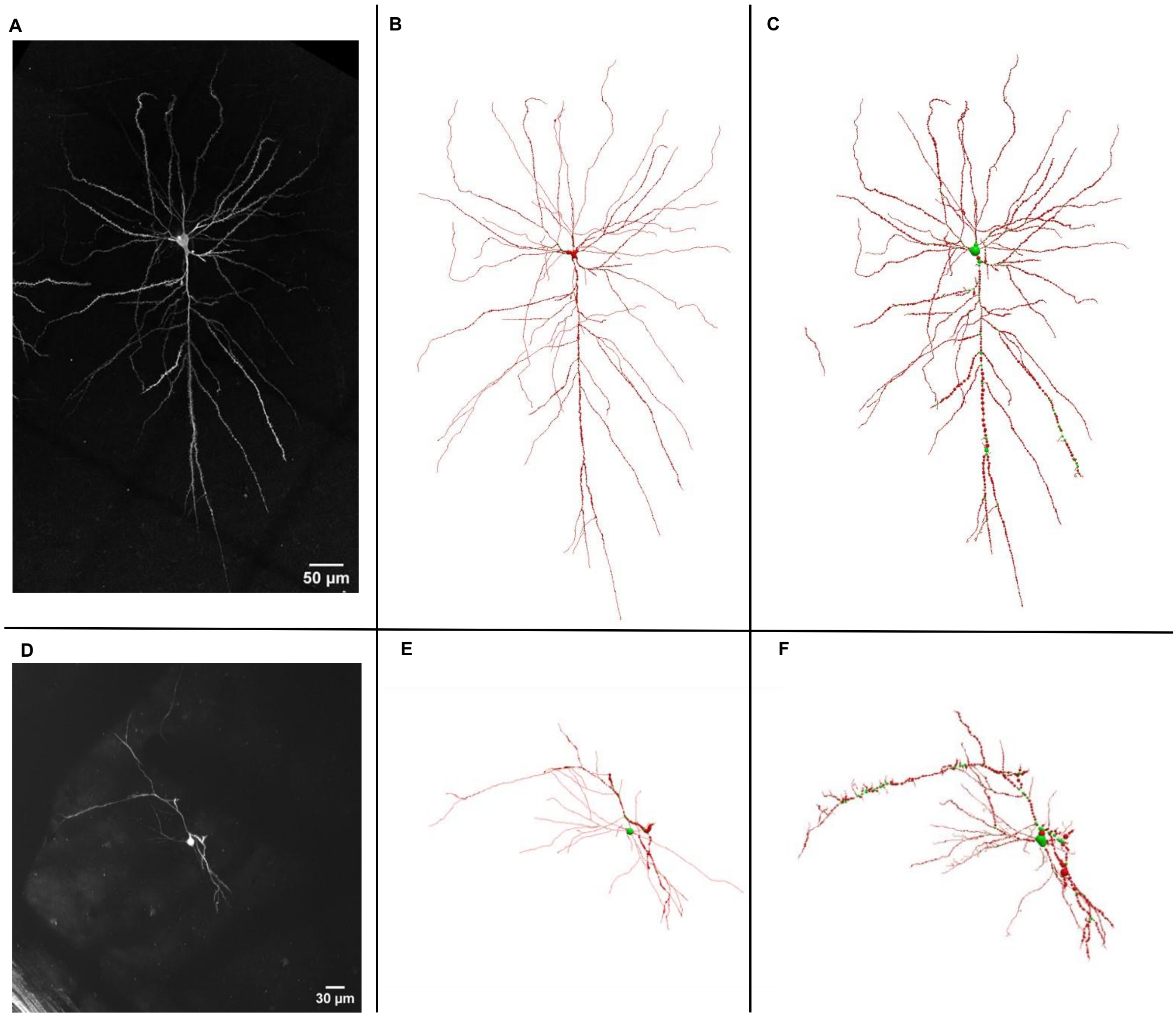

Supplementary Figure 8 Benchmark of SENPAI using the BigNeuron resource. A) Exemplary dataset (m16_cing_1_9_cropped_neurona.v3dpbd, human pyramidal cell labeled with Lucifer Yellow and acquired through confocal microscopy, resolution 0.24 µm x 0.24 µm x 0.42 µm) from Benavides-Piccione et al.[7] available from the BigNeuron gold166 standard[8], along with **B)** gold-standard (GS) segmentation of neuron in panel A and **C)** SENPAI segmentation. **D)** Exemplary dataset (neuron2.v3dpbd, excitatory

neuron from auditory cortex, layer 2/3, acquired through 2-photon microscopy, resolution 0.27 µm x 0.27 µm x 1 µm [9]) from the mouse cultured cell Cambridge dataset along with **E)** gold-standard (GS) segmentation of neuron in panel D and **F)** SENPAI segmentation.

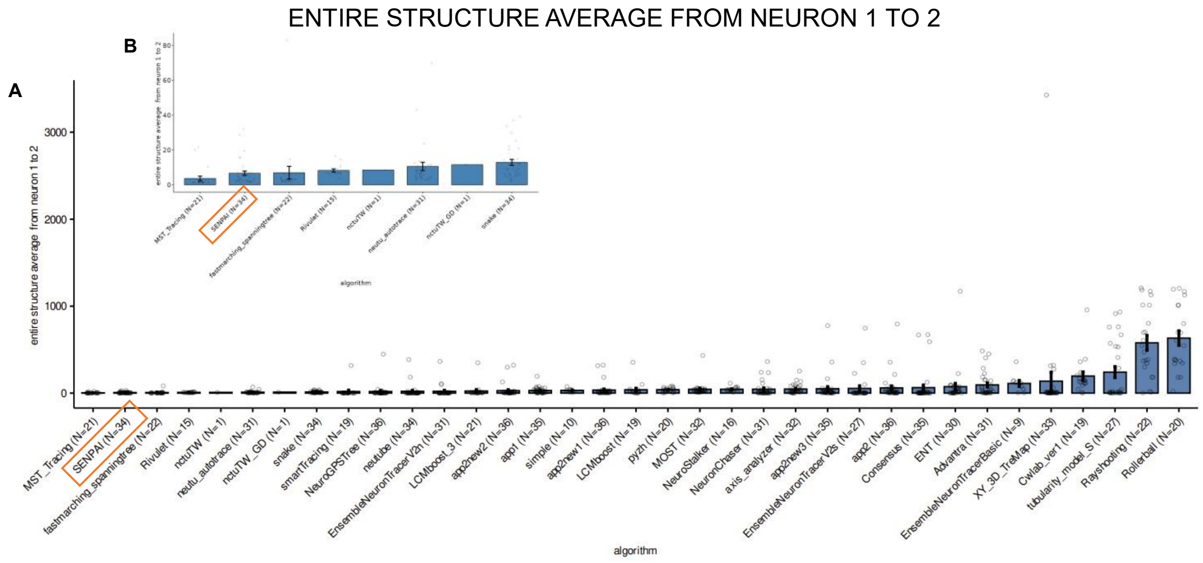

Supplementary Figure 9 Benchmark of SENPAI using the BigNeuron resource. A) Entire structure average from neuron 1 (GS) to neuron 2 (algorithm) benchmark (SENPAI's ranking 2/35). B) Detailed zoom of the first 8 ranked algorithms. Source data are provided as a Source Data file.

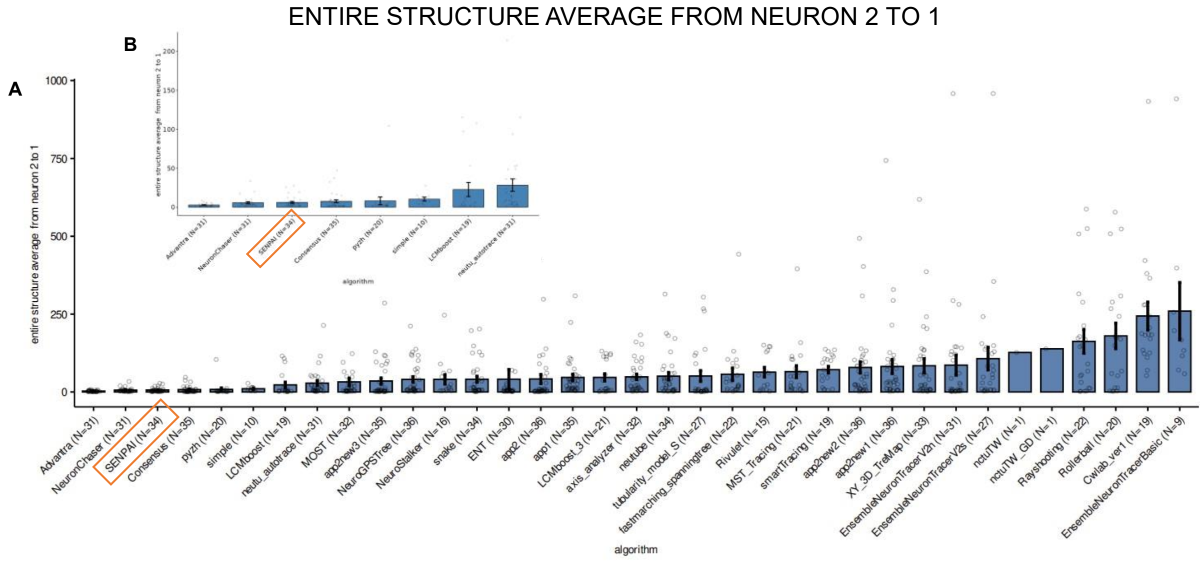

Supplementary Figure 10 Benchmark of SENPAI using the BigNeuron resource. A) Entire structure average from neuron 2 (algorithm) to neuron 1 (GS) benchmark (SENPAI's ranking 3/35). B) Detailed zoom of the first 8 ranked algorithms. Source data are provided as a Source Data file.

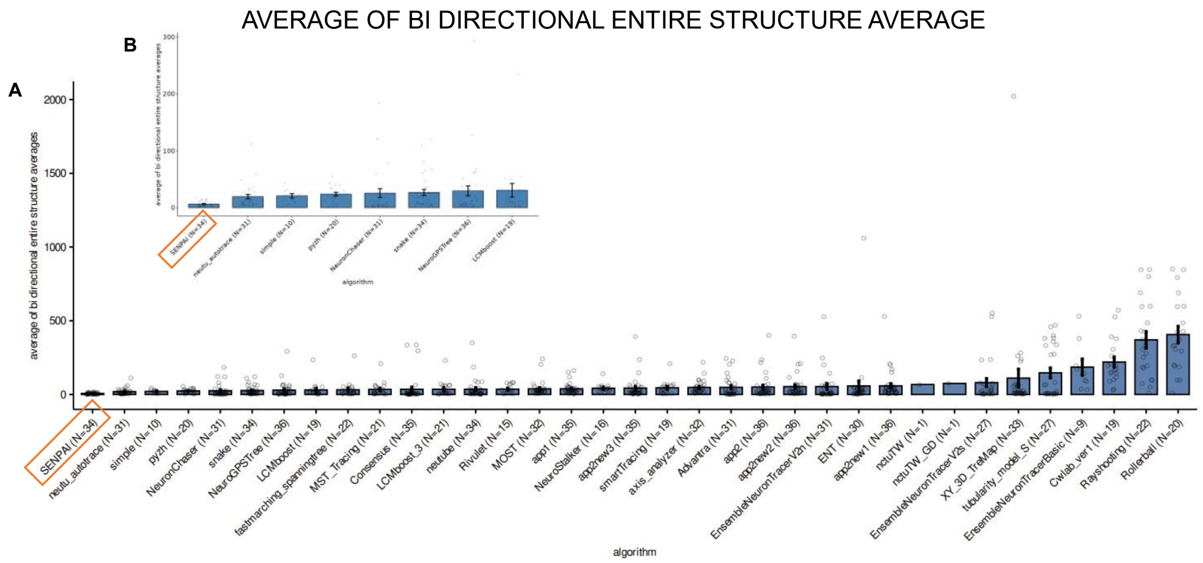

Supplementary Figure 11 Benchmark of SENPAI using the BigNeuron resource. A) Average of bidirectional entire structure averages with respect to the GS benchmark (SENPAI's ranking 1/35). B) Detailed zoom of the first 8 ranked algorithms. Source data are provided as a Source Data file.

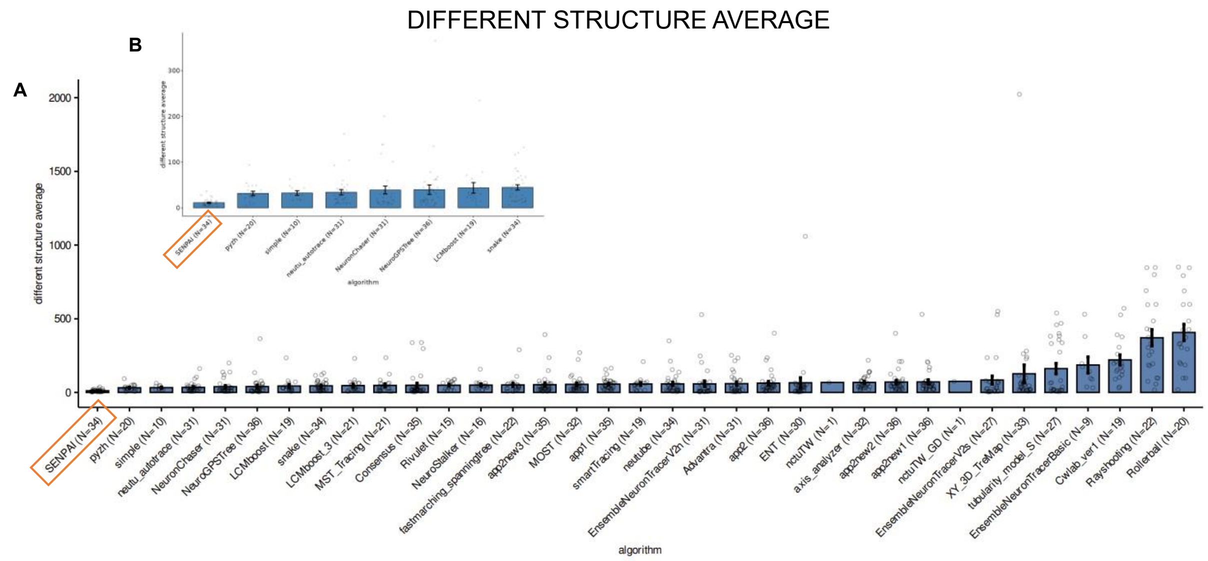

Supplementary Figure 12 Benchmark of SENPAI using the BigNeuron resource. A) Different structure average benchmark (SENPAI's ranking 1/35). B) Detailed zoom of the first 8 ranked algorithms. Source data are provided as a Source Data file.

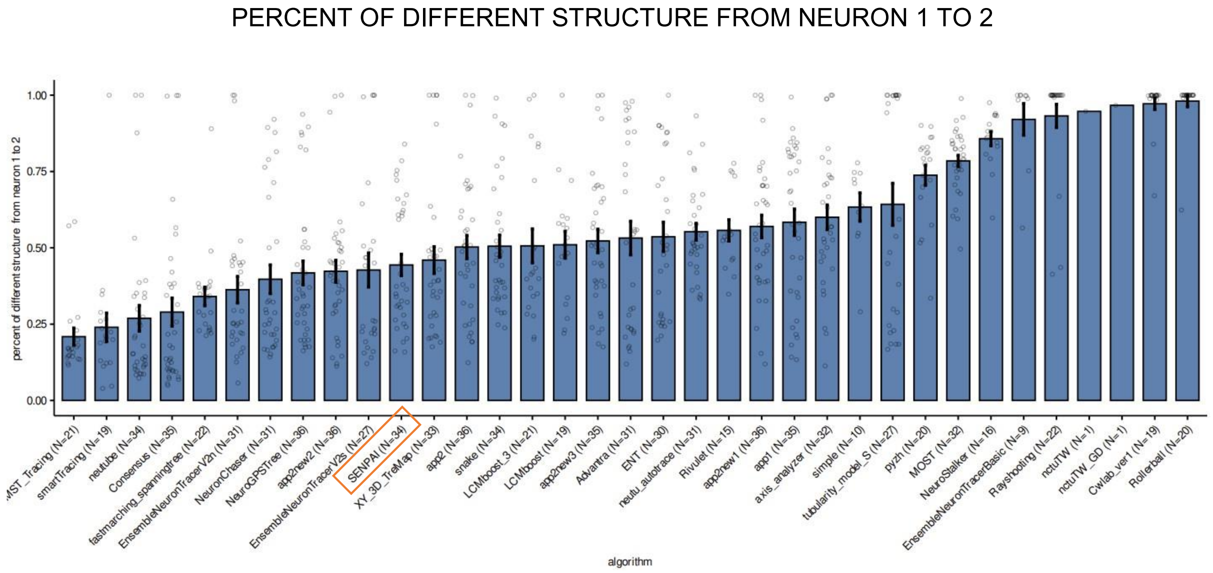

Supplementary Figure 13 Benchmark of SENPAI using the BigNeuron resource. Percent of different structure from neuron 1 (GS) to neuron 2 (algorithm) benchmark (SENPAI's ranking 11/35). Source data are provided as a Source Data file.

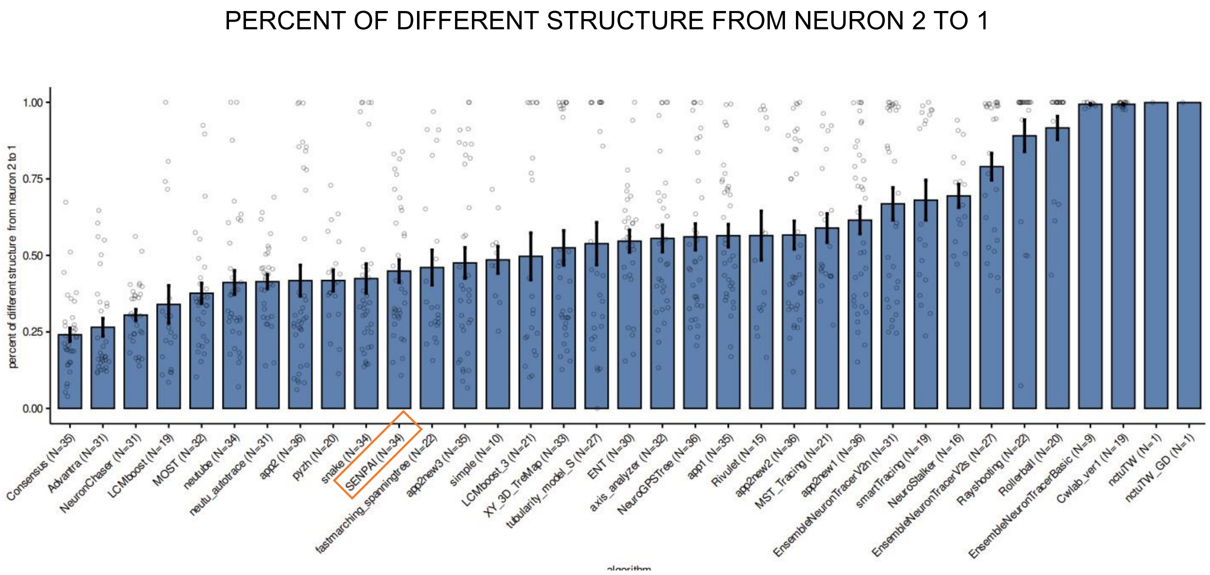

Supplementary Figure 14 Benchmark of SENPAI using the BigNeuron resource. Percent of different structure from neuron 2 (algorithm) to neuron 1 (GS) benchmark (SENPAI's ranking 11/35). Source data are provided as a Source Data file.

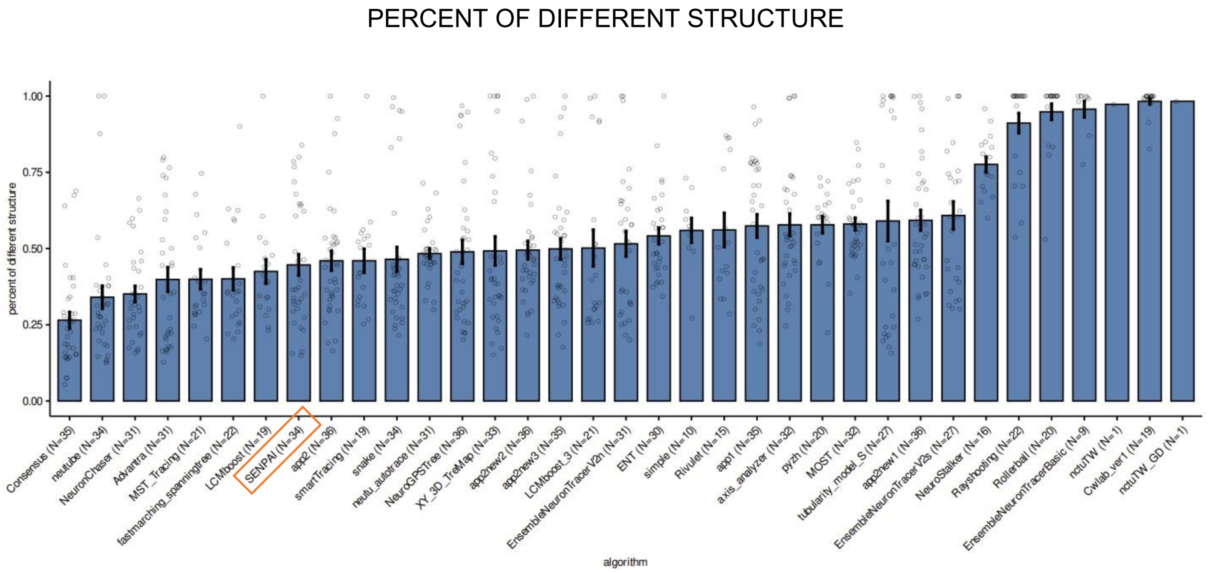

Supplementary Figure 15 Benchmark of SENPAI using the BigNeuron resource. Percent of different structure benchmark (SENPAI's ranking 8/35). Source data are provided as a Source Data file.

AGGREGATED DISTANCE

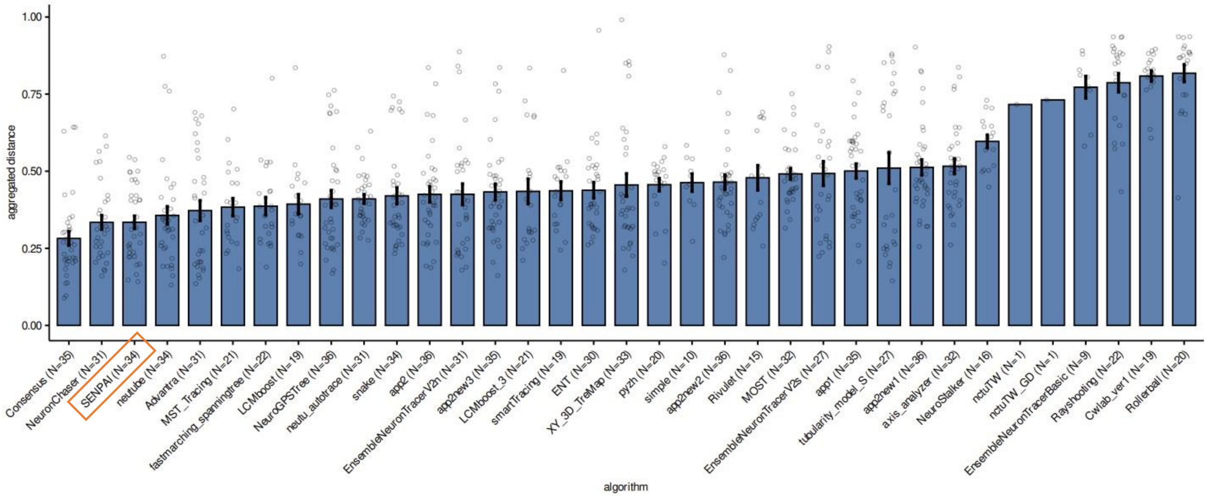

Supplementary Figure 16 Benchmark of SENPAI using the BigNeuron resource. Aggregated distance benchmark (SENPAI’s ranking 3/35). Source data are provided as a Source Data file.

Supplementary Note 11. SENPAI against state-of-the-art algorithms on the NeuroGPS test dataset

We tested SENPAI on the image stack downloadable from https://sourceforge.net/projects/neurogps-tree/files/ , as reported in the Code Availability Statement in Quan et al. 2016[10]. In Supplementary Figure 17, panels relative to Manual, NeuroGPS-Tree, Open-Snake and NeuroStudio tracings were imported from Figure 2, panel C in Quan et al. 2016[10].

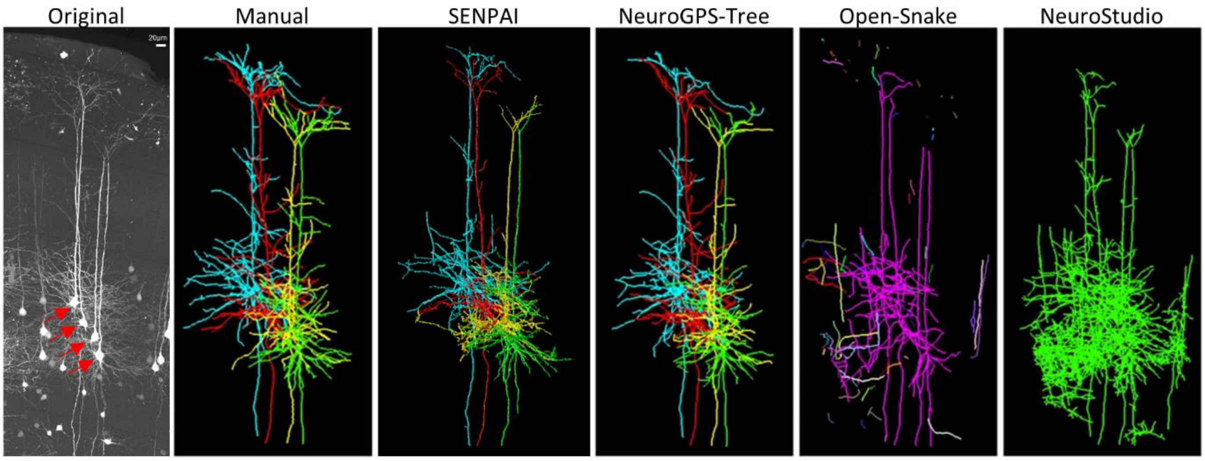

Supplementary Figure 17: Reconstructions performed with different algorithms on the test data provided with the NeuroGPS reference paper[10]. From left to right: Original data (red arrows indicate the segmented neurons), Manual, SENPAI, NeuroGPS-Tree, Open-Snake and NeuroStudio segmentations. Reproduced and adapted with permission from Figure 2, panel C, in [10].

Supplementary Note 12. Reconstructions on non-clarified samples: whole pyramidal cells

We tested SENPAI on two 3D stacks of cultured hippocampal pyramidal cells (Supplementary Figure 18). The sample was processed and imaged at INSERM. The pyramidal cells were transfected with a GFP targeted to plasma membrane, and then revealed with anti-GFP-A594 antibody in red. We did a counterlabeling, to verify which cell type were transfected. The inhibitory GABAergic interneurons are in cyan labeled with an anti GABA synthesis enzyme called GDA65-GAD67. In our first experiments, only pyramidal excitatory cells (not labelled in cyan) were transfected.

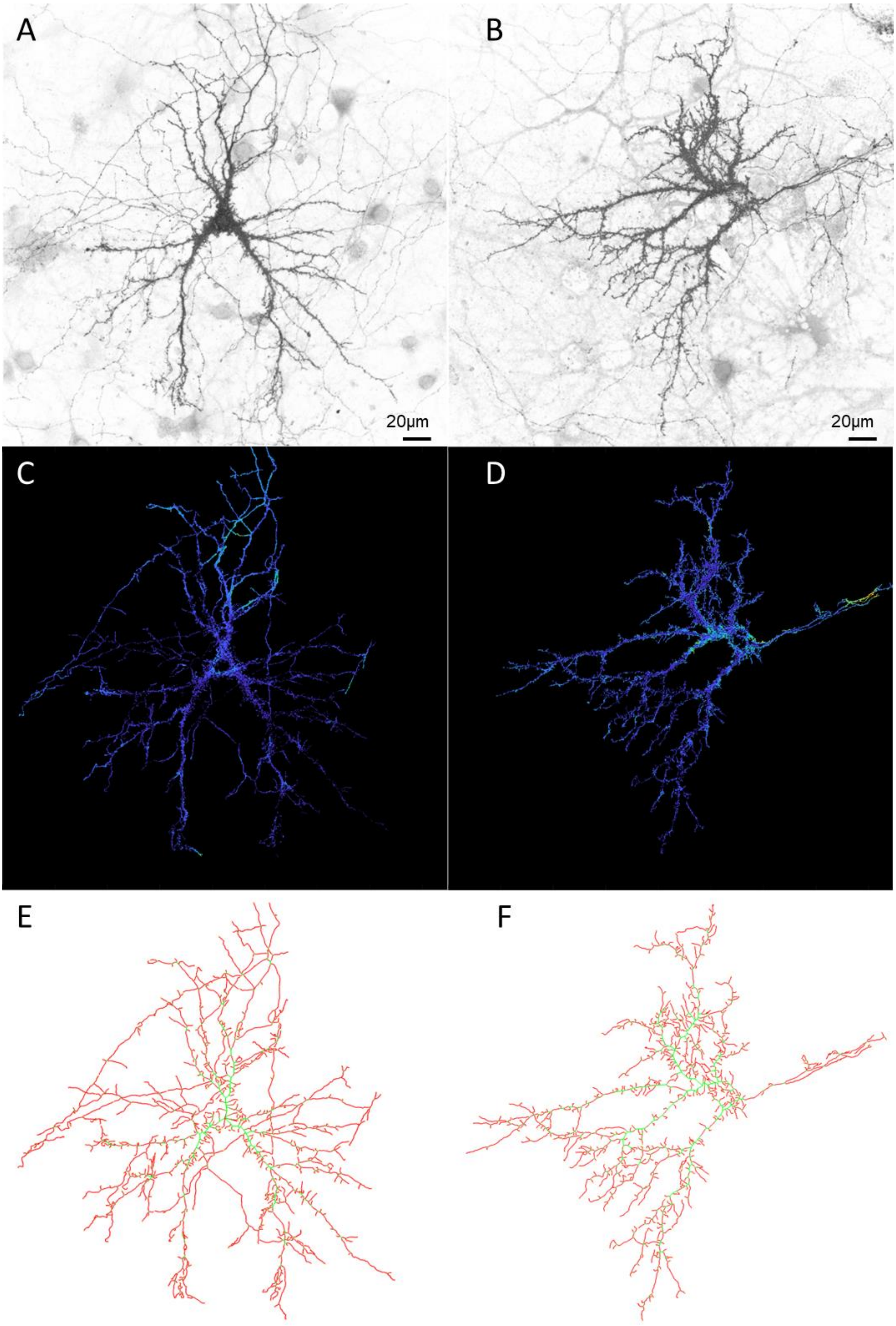

Supplementary Figure 18: SENPAI's outcome on two 3D stacks of cultured hippocampal pyramidal cells (pixel size 91,41 nm x 91,41 nm x 280nm). A-B) Original Image Maximum projection for the GFP channel. **C-D)** segmentation obtained with SENPAI with depth color-coded (cold colors indicate deeper planes). **E-F)** Skeletonizations of SENPAI segmentations produced with NeuTube.

Supplementary Note 13. Reconstructions on non-clarified samples: focus on spines of hippocampal pyramidal cells

For one of the samples presented on section 12 (see Supplementary Figure 18), two higher-resolution images were acquired, including in their field of view one same region (Supplementary Figure 19). Labeling was performed with membrane GFP instead of cytosolic GFP, gaining specificity for spine necks. The two images were acquired with confocal (Supplementary Figure 19.C) and 3D STED (Supplementary Figure 19.D) techniques. We report in panels E and F of Supplementary Figure 19 the segmentations obtained with SENPAI, color-coded for depth.

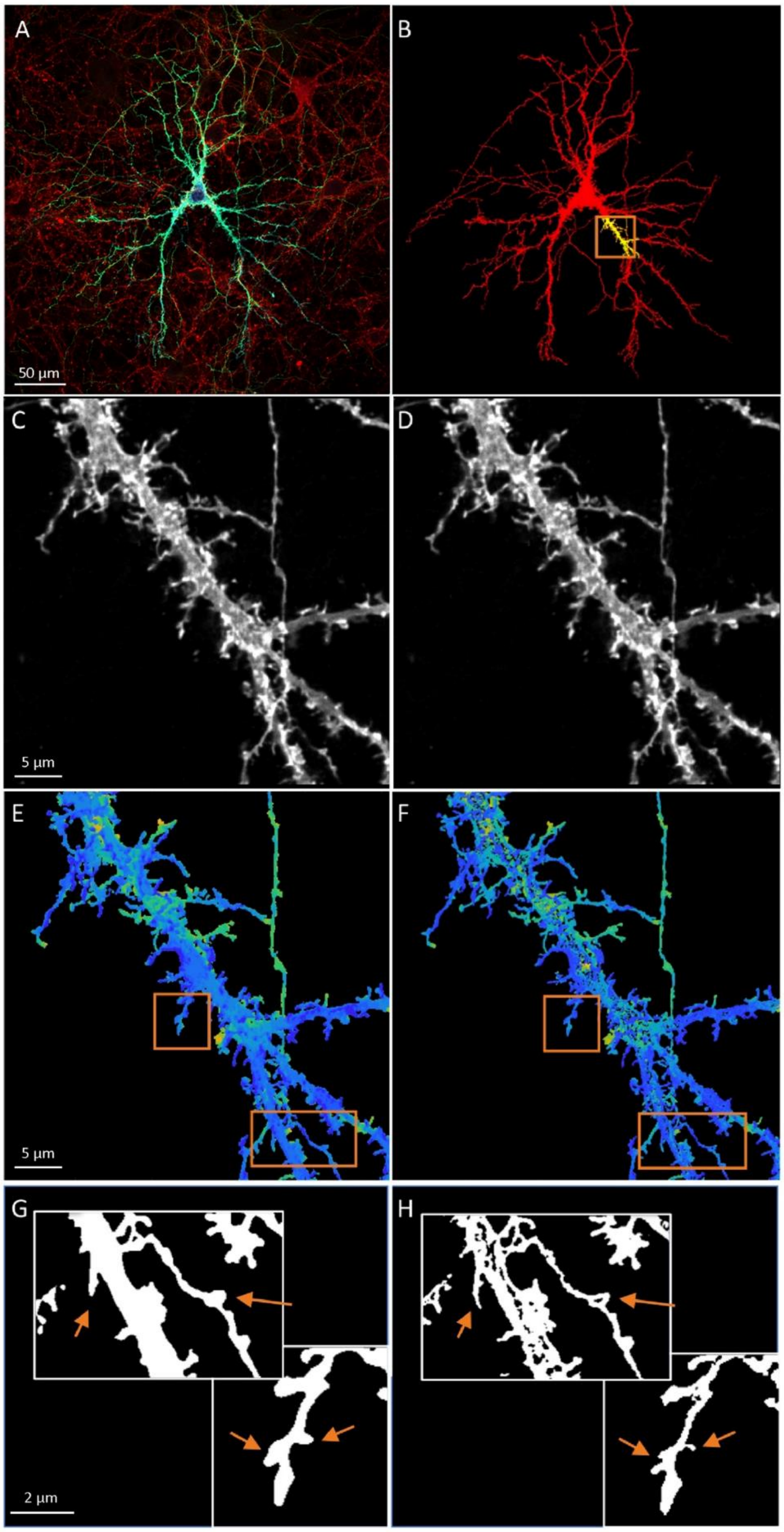

Supplementary Figure 19: SENPAI's outcome on high resolution images of dendritic spines. A) Original data, rat pyramidal hippocampal neuron transfected with a CMV-membrane GFP plasmid. GFP was then amplified with a GFP immunochemistry and revealed with Alexa594. **B)** Global SENPAI reconstruction of A). **C)** Region of A acquired with confocal at 93x **D)** Region of A acquired with STED at 93x. **E)** SENPAI reconstruction (depth color-coded) on confocal data in C) **F)** SENPAI reconstruction (depth color-coded) on STED data in D). **G)** In-depth detailed zoom of SENPAI reconstruction on confocal data 93x slice. **H)** In-depth detailed zoom of SENPAI reconstruction on STED data 93x (slice). Fine details are shown by orange arrows.

Supplementary Note 14. Visualization of reconstructions on 40x images

We provide in Supplementary Figure 20 an additional image for the reconstructions on selected dendritic branches from the 93x image.

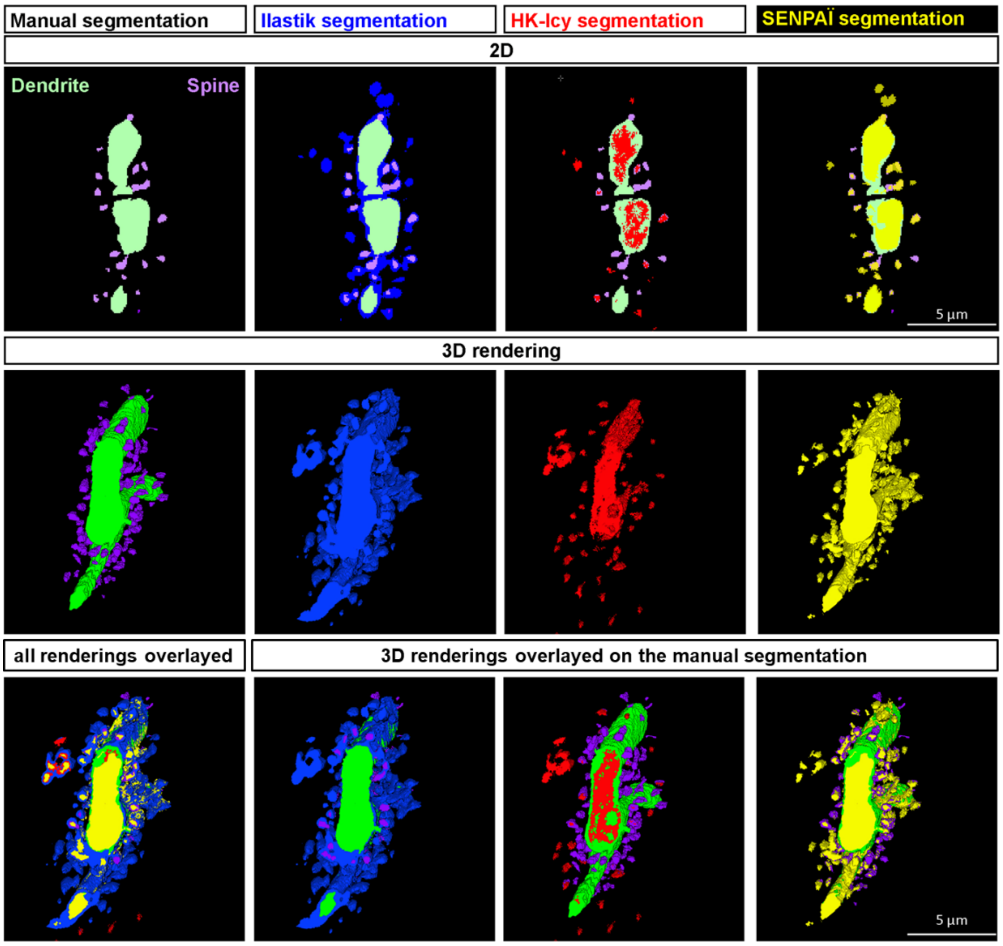

Supplementary Figure 20: 2D and 3D renderings of manual, SENPAI, HK-Icy and Ilastik reconstructions for the dendritic branches displayed in Figure 6 of the manuscript. top) 2D renderings: for the manual segmentation, spines (purple) and the dendritic trunk (green) are visualized with different colors; for the three algorithms, the 2D rendering of Ilastik (blue), HK-Icy (red) and SENPAI (yellow) are overlayed on the manual segmentation. **middle)** 3D renderings using the same colors as in the top row. 3D renderings of the reconstructions obtained with the three algorithms are displayed with no overlay. **bottom)** 3D renderings using the same colors as in the top row. All segmentations are displayed together on the left panel. On the three other panels, the segmentation obtained with each algorithm is rendered together with the manual segmentation.

Supplementary Note 15. Time to run the algorithm

On our test_data.tif dataset, i.e., a 512-by-512-by-143 voxels 3D stack:

- RAM 16 GB, processor Intel® Core™ i7-10750H CPU @ 2.60GHz: segmentation+parcellation in 804 seconds.
- RAM 16 GB, processor Intel® Core™ i7-1165G7 CPU @ 2.80GHz: segmentation+parcellation in 1000 seconds.
- RAM 32 GB, processor Intel® Core™ i7-12700K CPU @ 3.60GHz: segmentation+parcellation in 360 seconds.

Supplementary References

1. Magliaro, C., Callara, A. L., Vanello, N. & Ahluwalia, A. Gotta Trace 'em All: A Mini-Review on Tools and Procedures for Segmenting Single Neurons Toward Deciphering the Structural Connectome. *Front. Bioeng. Biotechnol.* **7**, (2019).

2. Liu, Y., Wang, G., Ascoli, G. A., Zhou, J. & Liu, L. Neuron tracing from light microscopy images: automation, deep learning and bench testing. *Bioinformatics* **38**, 5329–5339 (2022).

3. Chen, R., Liu, M., Chen, W., Wang, Y. & Meijering, E. Deep learning in mesoscale brain image analysis: A review. *Comput. Biol. Med.* **167**, 107617 (2023).

4. Vormberg, A., Effenberger, F., Muellerleile, J. & Cuntz, H. Universal features of dendrites through centripetal branch ordering. *PLOS Comput. Biol.* **13**, e1005615 (2017).

5. Manubens-Gil, L. *et al.* BigNeuron: a resource to benchmark and predict performance of algorithms for automated tracing of neurons in light microscopy datasets. *Nat. Methods* **20**, 824–835 (2023).

6. Peng, H., Bria, A., Zhou, Z., Iannello, G. & Long, F. Extensible visualization and analysis for multidimensional images using Vaa3D. *Nat. Protoc.* **9**, 193–208 (2014).

7. Benavides-Piccione, R., Fernaud-Espinosa, I., Robles, V., Yuste, R. & DeFelipe, J. Age-Based Comparison of Human Dendritic Spine Structure Using Complete Three-Dimensional Reconstructions. *Cereb. Cortex* **23**, 1798–1810 (2013).

8. Peng, H., Ruan, Z., Long, F., Simpson, J. H. & Myers, E. W. V3D enables real-time 3D visualization and quantitative analysis of large-scale biological image data sets. *Nat. Biotechnol.* **28**, 348–353 (2010).

9. Peter, M. *et al.* Transgenic Mouse Models Enabling Photolabeling of Individual Neurons In Vivo. *PLoS One* **8**, e62132 (2013).

10. Quan, T. *et al.* NeuroGPS-Tree: automatic reconstruction of large-scale neuronal populations with dense neurites. *Nat. Methods* **13**, 51–54 (2016).
