# Supplementary material for: A modular framework for multi-scale tissue imaging and neuronal segmentation
Source: Nat Commun. 2024 May 22;15:4102. doi: 10.1038/s41467-024-48146-y (PMC11111705; doi:10.1038/s41467-024-48146-y)
Supplement: Supplementary file 3 — Description of Additional Supplementary Files [file 41467_2024_48146_MOESM3_ESM.pdf]

Title: Supplementary Movie 1
Description: 3D rotating views of acquired standard- and super-resolution datasets

Title: Supplementary Movie 2
Description: 3D rotating views of a dense 40x confocal stack (red) and one segmented neuron (green)
